# A multiplayer game model to detect insiders in wireless sensor networks

**DOI:** 10.7717/peerj-cs.791

**Published:** 2022-01-20

**Authors:** Ioanna Kantzavelou, Leandros Maglaras, Panagiotis F. Tzikopoulos, Sokratis Katsikas

**Affiliations:** 1 University of West Attica, Athens, Greece; 2 Cyber Technology Institute, De Montfort University Leicester, Leicester, UK, United Kingdom; 3 Banking and Financial Management, University of Piraeus, Piraeus, Greece; 4 Norwegian University of Science and Technology, Gjøvik, Norway

**Keywords:** Intrusion detection, Game theory, Wireless sensor networks, Multiplayer game

## Abstract

Insiders might have incentives and objectives opposed to those of the belonging organization. It is hard to detect them because of their privileges that partially protect them. In Wireless Sensor Networks (WSNs), significant security issues arise, including compromised nodes by insiders that disrupt the normal network operation. Immediate defensive actions to isolate malicious nodes would mitigate any related impacts. A multiplayer game model is proposed as a solution to the problem of insider attacks in WSNs, the Game of Wireless Sensor Networks (GoWiSeN). It is an imperfect information game, formulated with the use of non-cooperative game theory, holding the assumption that all players are rational. The model consists of several Local Intrusion Detection Systems (LIDSs), which are located to different nodes and communicate with a Global Intrusion Detection System (GIDS). Each LIDS gives suggestions whether the monitoring node is trusted or not. The game is being played between a potential attacker, the nodes and the GIDS. The GIDS is responsible for making a final decision and for isolating a compromised node in case of an internal attack. The theoretical model represents these interactions in an extensive form game. The formal elements of the game are specified, the outcomes of the game are quantified by first specifying players’ preferences, and then, by using the von Neumann-Morgenstern utility function, and payoffs are obtained. The game is constructed and solved, by locating NE in pure and mixed strategies. Experimental evaluations conducted on real network datasets, using IDSs of different capabilities, simulate special cases and compromised nodes in a WSN, verify the model efficiency, and show how the game should be played.

## Introduction

Wireless Sensor Networks (WSNs) were introduced some years ago as a new technology that combines wireless communication (Alcaraz, 2019), computation, and sensing (Stankovic, 2008). The great range of applications has made WSNs very popular and the need for simple and familiar interactions more essential than ever. As part of pervasive computing environments, WSNs raise fundamental security issues. Attacks on sensor networks routing (novel attacks-sinkhole and HELLO floods) have imposed new design for secure routing protocols (Karlof & Wagner, 2003). Detection of masquerade attacks on WSNs requires lightweight techniques with respect to important WSN properties, like coverage, connectivity, data aggregation and specific communication patterns (Bhuse & Gupta, 2006). Such characteristics have generated special attacking methods to WSNs, as the sleep deprivation attack (Pirretti et al., 2005), the time synchronization attack (Manzo, Roosta & Sastry, 2006), and the selective forwarding attack (Yu & Xiao, 2006). Several taxonomies of attacks on WSNs has been proposed in Han et al. (2005), Nawir et al. (2016).

Intrusion Detection plays an active role in cybersecurity and many different technologies, tools, and approaches have been used in conjunction with it, as blockchain technology (Agarwal et al., 2021), machine learning (Dua & Du, 2016), and fuzzy logic (Elhag et al., 2015). As an important area of research, Intrusion detection has been applied in WSNs to enhance their security, despite the fact that it is used as a second line of defense. Classical detection techniques have been employed over lightweight Intrusion Detection Systems (IDSs), like the anomaly intrusion detection technique in Bhuse, Gupta & Al-Fuqaha (2007), to detect and deter attacks that affect the normal and uninterruptible operation of WSNs.

One of the main security problems in WSNs is the problem of compromised nodes, as defined in Zhang, Yu & Ning (2006). Although cryptographic mechanisms are used to protect sensor networks from masquerading attacks, attackers might compromise a node by stealing a key, and introducing afterwards faulty data from this compromised node. There are two approaches to address this problem; either distinguish faulty data from real data, or detect which node is the compromised node and exclude it from the network. The first approach has been mainly employed by other related works, but without significant results. Abdalzaher et al. (2016) introduced the concept of evolutionary game using a group policy/authentication method to resist intelligent attacks which do not use pure strategies.

Since a great number of attacks against sensor network routing originated by outsiders can be evaded by the use of authentication and encryption mechanisms (Karlof & Wagner, 2003), insider attacks are the most challenging and demanding to be counteracted. The proposed work addresses the problem of detecting insider attacks in WSNs, by taking the advantages of intrusion detection when incorporating findings of game theory. The current manuscript is an extended version of the previously published article in Kantzavelou, Tzikopoulos & Katsikas (2013). Compared to the previous publication, it incorporates evaluation results of the IDSs under a realistic Internet of Things (IoT) dataset, which affect the players’ payoffs, analyses specific cases where nodes belong to more than one clusters, and discusses the situation where a Local Intrusion Detection System (LIDS) is compromised.

In this paper, a game model between a potential attacker, and the IDSs used in a WSN is proposed. The potential attacker is an internal user of the system, who acts normally most of the times, but occasionally attacks the system, by compromising a node of the WSN.

Following Osborne’s and Rubinstein’s dimensions (Osborne & Rubinstein, 1994), the *player*, the *plan of actions*, and the *information*, upon which three divisions of game theoretic models are based, a *non-cooperative* game theoretic model is constructed, in *extensive form* that allows each player to think about his plan of actions whenever he plays, formulating the sequential moves of interactions, with *imperfect information*.

The under construction game is named *GoWiSeN* as an abbreviation of *‘Game of Wireless Sensor Networks’*. According to prescriptive game theory, theoretical examination of the constructed game, allows us to determine how players should play it, and to recommend strategies. As a result, it is possible to give advice that helps players to make better decisions.

The main contributions of the article are summarized in the following:

- A three player non-cooperative game is modeled, to study the interactions between an insider and the IDSs used in a WSN (Section The Game).
- The solution of the game by locating Nash Equilibria in mixed strategies is provided (Section Solution of the Game).
- Evaluation of the model is conducted using a dataset from realistic network environment that includes normal and botnet traffic (Section Experimental Evaluations).
- Discussion on special cases of nodes that belong to overlapping clusters is given (Section Special Cases).
- The situation where a LIDS is compromised is examined (Section Special Cases).

The paper has been organized in nine sections. In the Related Work section, review related works and discussion against the proposed work is provided. The architecture of the proposed model is illustrated in The Architecture of the Model section. The game that models the interactions between the different parties of this architecture is constructed and solved in The Game and Solution of the Game sections. A complete case study is presented in the A Case Study section and two different scenarios are described to explain the implementation and functioning of the game model. In the Experimental Evaluations simulated results on a realistic dataset are presented. In the Special Case section, two special cases are discussed with regards to how they can be modeled and analyzed. Finally, in the Conclusion and Future Work section, our research work is summarized by evaluating the model and its operation, and suggesting future directions.

## Related work

The problem of detecting attackers in WSNs has been addressed by various approaches. The ultimate aim is the discrimination between compromised nodes and normal ones. A malicious node should be isolated as soon as identified, because it has no trust value and prohibits the normal network operation. Among the large number of constraints inherited in WSNs, the most important are; the low computational capability, and the limited recourses regarding memory and energy. In addition to these, security issues enhance, in many ways, their vulnerabilities and make them easy targets.

A number of detection engines proposed as a solution to this problem are presented in Sharma & Athavale (2019), through the discussion of their corresponding research works that incorporate different tools, technologies and methods. Probabilistic models, game theoretic approaches, K-means clustering, artificial immune systems, distributed anomaly detection techniques, genetic algorithms and Random Neural Networks (RNN) are a sample of these solution approaches. The authors give valuable conclusions for the importance of detection in WSNs and the possible architectures that might be incorporated in constructing dedicated IDSs for these type of networks.

Game Theory acts as a set of tools to model interactive situations. Camerer describes it as the answers to mathematical questions regarding what players with ranging rationality will do in the future (Camerer, 2003). Game theoretic approaches consequently have gained favor in many research works the last few years (Naseer et al., 2021), many related to cybersecurity and specifically to intrusion detection. Kiennert et al. (2018) have investigated different solutions that might improve the efficiency of intrusion detection systems with the use of game theory.

Another survey on game theoretic approaches used in WSNs concentrates on three main problems; sensor’s energy efficiency, network security, and pursuit-evasion games (Machado & Tekinay, 2008). The majority of the works described tackle the sensor’s energy efficiency problem, though network security and pursuit-evasion games are very significant too. Examining the problem of security, two types of threats are mentioned, the external attacker, and the malicious nodes within the sensor network. Another interesting survey on game theoretic approaches for WSNs can be found in Shi et al. (2012).

The problem of compromised nodes has been addressed by Zhang, Yu & Ning (2006). They propose an application-independent framework for identifying compromised sensor nodes, and they develop alert reasoning algorithms for this identification. Their technical approach uses an observer model.

LIDSs have been defined in an adapted architecture for an intrusion detection system for manets in Albers et al. (2002). In another work Ma et al. (2006), take advantages of LIDSs to have a tradeoff among the security of WSN and communication overhead.

To address security problems mentioned above, Subba, Biswas & Karmakar (2018) propose in, a game theoretic multi layered intrusion detection framework for WSNs. The proposed framework uses specification rules and a lightweight anomaly detection module to identify malicious sensor nodes. The framework models the interactions between the IDS and a node as a two player non-cooperative Bayesian game, which guides the IDS how to choose proper strategies based on the Bayesian Nash Equilibrium. The game is supported by two mechanisms that strengthen cooperation, the Shapley Value and the Vickery–Clark–Grooves (VCG) mechanism.

Han et al. (2019) propose in a game theoretic solution approach to solve two problems in WSNs; the limited resources and the efficiency in detecting malicious nodes. A non-cooperative, complete-information, static game model is constructed and solved using the Nash equilibrium approach, to isolate the optimal defense strategies that balances the system’s detection efficiency and energy consumption. In addition, an autoregressive model is built as a prediction model to locate the attacker’s target node.

In Wang et al. (2016), focus on the area of Intrusion Detection to solve security problems in Cyber-Physical Embedded Systems (CPESs), admitting that existing security mechanisms cannot directly be applied to Embedded Sensor Networks (ESNs). They propose a new attack-defense game model to detect malicious nodes and choose to play the game repeatedly. The IDS takes into consideration two important parameters; error detection and missing detection. The proposed model achieves to reduce energy consumption and increase the detection rate, and thus enhance ESNs security using this active defense approach.

The problem of IoT heterogeneous devices connected to untrusted networks is discussed in Sedjelmaci, Senouci & Taleb (2017). The authors expose the advantages gained by the combination of the anomaly and the misuse detection techniques in a single detection engine, which reduces the high false positive and false negative alarms respectively. But, this simultaneous activation in low-resource IoT devices could generate a high-energy consumption. To overcome this drawback, Sedjelmaci, Senouci & Taleb (2017) propose a game theoretic approach that activates anomaly detection technique only when a new attack’s signature become evident. The anomaly detection technique models the normal behavior of a node through a learning algorithm, and when a new attack pattern appears, it models it following a set of rules. Moreover, a reputation model based on game theory supplements the aim of reducing false rates.

Bai et al. (2019) construct a game to model the attack and defense interactions between nodes and an IDS in WSNs. The aim is to determine the optimal defensive strategies an IDS should select in order to reduce energy consumption and improve detection efficiency. The scalability of the system is another problem addressed by the incorporation of the agent technology, which also leads to system fault tolerance enhancement.

The incorporation of trust management models in WSNs as an alternative security mechanism has been suggested in different research works (Han et al., 2014). They aim at detecting malicious attacks, secure routing, secure data aggregation, secure localization, or secure node selection. The common point in most of them is trust. Some research works that employ a trust model to detect malicious nodes in WSNs are discussed in the next paragraphs.

Wu et al. (2015) start by pointing out two WSN features, the openness feature imposed by the wireless connectivity and the inherent self-organization feature in WSNs, and both reveal a significant concern regarding the trust evaluation of network nodes. By accepting the difficulty in recognizing a node’s behavior and making a decision over it, they propose a new trust model based on fuzzy theory and revised evidence theory, to detect nodes that have anomaly behavior. It is a trust-based anomaly detection model that aims at verifying the normal operation of the network by identifying malicious nodes in it (Wu et al., 2015).

A hybrid IDS for WSNs that combines the anomaly and the misuse detection technique is presented in Singh, Singh & Singh (2017). The proposed system is based on fuzzy rule sets along with the Multilayer Perceptron Neural Network and addresses three types of attacks, the Sybil attack, the wormhole attack, and the hello flood attack, by adopting specific algorithms for each of them. Experimental results show the effectiveness of the presented IDS in detecting malicious nodes in a WSN, with high true positive rate and low false positive rate.

A trust evaluation model based on an entropy weight assignment method is proposed in Yin & Li (2019) for the detection of malicious nodes in WSNs. The work addresses attack types related to packet dropping or packet modification by incorporating trust indicators and estimates trust values that reflect adjacent nodes behavioral information. The use of an entropy weight method improves the evaluation procedure and the trust concluding results, which lead to the detection of malicious nodes.

Another method to detect insiders in WSN is a trust-based mechanism presented in Meng, Li & Kwok (2013). A Bayesian model is deployed in a detection mechanism to isolate malicious nodes and permit the benign ones to continue the normal network operation. This trust model allows the establishment of trust values in an hierarchical structure, which reduces the network traffic and facilitates detection. The authors conclude that the proposed method shows experimentally that a Bayesian trust model is a suitable solution for the detection of malicious nodes in WSNs comparing to their corresponding work in wired networks.

An alternative approach to build a detection model, adequate for WSN, with a novel nonparametric Bayesian method is proposed in Alhakami et al. (2019). This method works with no need to specify parameters such as the number of clusters to detect both known and unknown attacks. A Bayesian-based MCMC inference for infinite bounded generalized Gaussian mixture models is used to learn the event patterns. Experimental evaluation results show the efficiency of the proposed method to detect a few types of attacks.

Another trust model based on clustering is proposed in Jaint et al. (2018). A weighted trust method is examined in a WSN that consists of a base station, a number of sensor nodes divided into some clusters, a node of each cluster selected to be the head of it, and a forward node that transmits aggregated data to the base station. The method aims at malicious node detection. Different scenarios regarding the clustering structure show that the detection time is less, the accuracy and the scalability are better, and the propagation time is less, when clustering includes multiple cluster heads with non-overlapping grid comparing to single cluster head without grid.

A detailed comparison of different trust models and the proposed GoWiSeN with respect to (1) method, (2) objective, (3) strengths, and (4) weaknesses is provided in Table 1.

**Table 1:** Comparison of trust models.

Strengths and Weaknesses					
Name	Trust model	Method	Objective	Strengths	Weaknesses
A game of wireless sensor networks (GoWiSeN)	A multiplayer game model, game theory	Game theoretic approach over IDS decisions.	Insider attacks in WSNs	Construction and solution of a three-player game with insiders. Game outcomes determined from a real network dataset. Examination of a compromised LIDS scenario.	Preferences reflect one type of attackers only.
Trust-based anomaly detection model (Wu et al., 2015)	Fuzzy trust model, Fuzzy Theory and Revised Evidence Theory	Trust-based anomaly detection technique with a weighting algorithm.	Detection of nodes that have anomaly behavior.	Energy efficient anomaly detection scheme towards practical application.	The evaluation node only accepts the packets from one-hop neighbors.
Advanced hybrid intrusion detection system (AHIDS) model (Singh, Singh & Singh, 2017)	Fuzzy trust model, Fuzzy Theory.	A hybrid IDS for WNS, Fuzzy rule sets along with the Multilayer Perceptron NN.	Detection of malicious nodes in WSNs.	Specific algorithms for each attack. Effective in detecting malicious nodes in WSNs.	Limited attack types. Extension to address more requires new specific algorithms.
Trust evaluation model (Yin & Li, 2019)	Entropy-based model.	Based on an entropy weight assignment method.	Detection of malicious nodes in WSNs.	The entropy-based weight assignment improves the objectivity of trust evaluation and obtains fast convergence rate.	Limited attack types. It addresses packet dropping and packet modification attacks.
Bayesian model (Meng, Li & Kwok, 2013)	Bayesian trust model.	Bayesian trust-based mechanism.	Detection of insiders in WSNs.	Trust values are established in an hierarchical structure to reduce network traffic and facilitate detection. More suitable for WSNs.	The work is developed at an early stage. The WSN is very limited.
Non-parametric Bayesian model (Alhakami et al., 2019)	Bayesian model.	Bayesian-based MCMC inference.	Detection of known and unknown atatcks in WSNs.	This method works with no need to specify parameters such as the number of clusters.	It addresses general network security detection problems rather than specific to WSNs.
Clustering trust model (Jaint et al., 2018)	Clustering trust model.	Cluster based weighted trust evaluation method.	Detection of malicious nodes in clustered WSNs.	Detection and propagation time are less, and the accuracy and the scalability are better with multiple cluster heads with non-overlapping grid.	Limited experimentation.

The proposed work focuses on the special case of internal attackers, and formulates a game model with three players, one assumed as a potential attacker (insider), an IDS monitoring a node of a WSN, named Local Intrusion Detection System (LIDS), and another one, located at the base station that cooperates with many LIDSs, named Global Intrusion Detection System (GIDS). The games played are between different LIDSs, the GIDS and an insider, as imposed by the model architecture in multiplayer game constructions. To the best of our knowledge, this is the only game-theoretic approach for intrusion detection in WSNs that constructs and solves multiplayer games for insiders.

Prior work has shown that, in the special case of insiders, Intrusion Detection can efficiently be employed in a game theoretic framework, to address significant security problems in WSNs (Kantzavelou, Tzikopoulos & Katsikas, 2013). Several other recent works study and take into account these preliminary results (Li et al., 2017; Ranaweera et al., 2019; Sánchez, Parra & Medina, 2019), and employ similar conceptual approaches. Li et al. (2017) recently designed an intrusion sensitivity-based trust management model for WSNs to defend against insider attacks. In their proposed work, each IDS evaluates the trustworthiness of others and automatically assigns the values of intrusion sensitivity with the use of machine learning techniques. The cooperation between different IDSs resembles the combined working of LIDSs and the GIDS, which was proposed in Kantzavelou, Tzikopoulos & Katsikas (2013). Ranaweera et al. (2019) also give an extension by applying traffic flow theory in order to identify anomalous data in vehicular networks, and provide reliable and consistent predictions against incorrect decisions. Finally, in Sánchez, Parra & Medina (2019), a game theoretic model for intrusion detection in WI-FI networks is presented.

## The architecture of the model

We have assumed a WSN with a large number of nodes, densely scattered in an area. This network consists of a set *C* of *n* clusters, *C* = {*C*_1_,*C*_2_,…,*C*_*n*_}. Each cluster covers a number of nodes, quantified as *m*_*i*_ for the *i*_*th*_ cluster, that communicate one another and each with a base station. Thus, the set of nodes for the *i*_*th*_ cluster is *N*_*i*_ = {*N*_*i*1_,*N*_*i*2_,…,*N*_*im*_}. Although the problem of positioning base stations in a sensor network is already under consideration (Bogdanov, Maneva & Riesenfeld, 2004), we have chosen the use of a single base station, for simplicity reasons.

**Figure 1:**
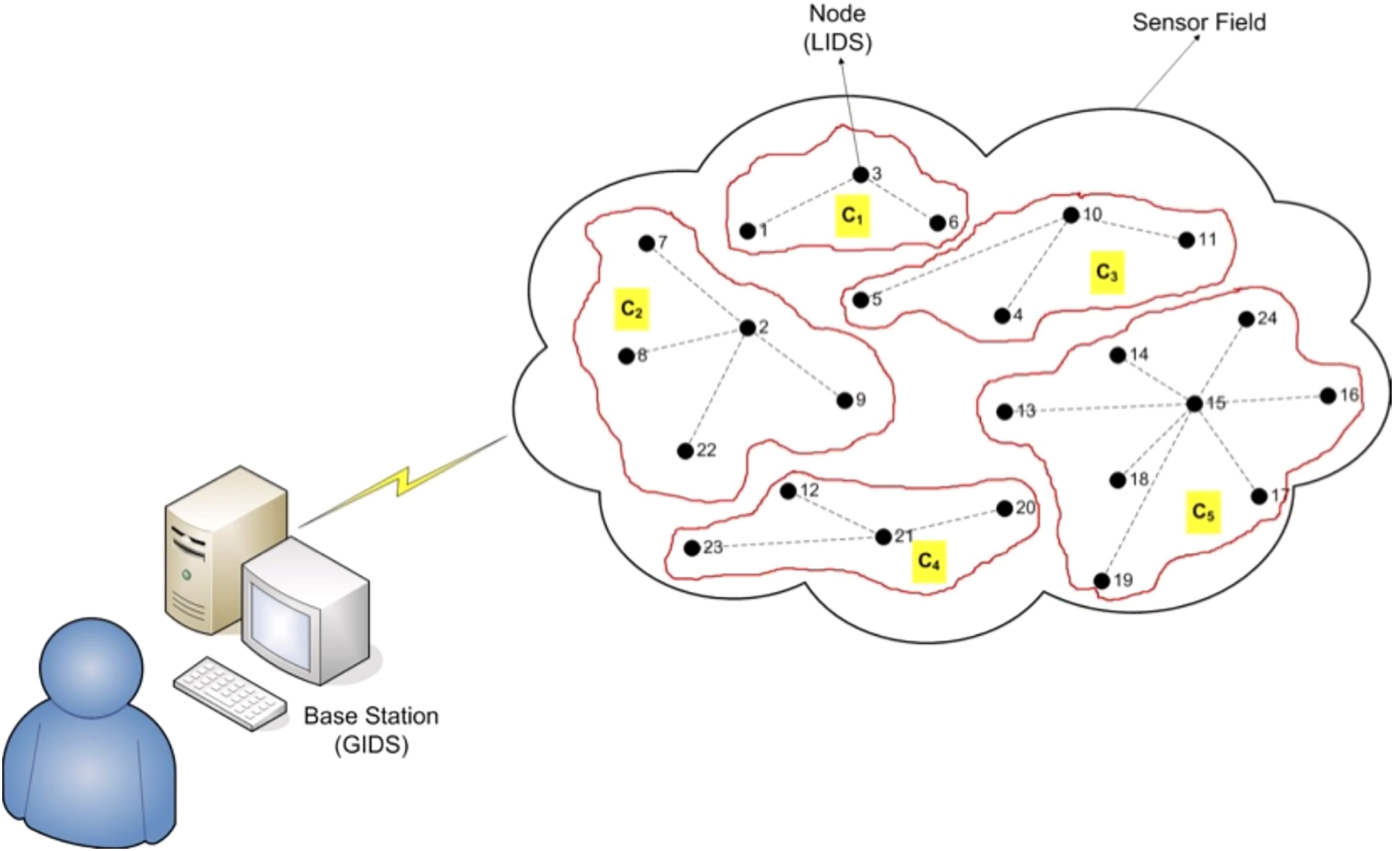
The architecture of the model.

In this network, there is a Global IDS, the GIDS, established on a separate machine, at the base station. This GIDS receives signs of suspicious or trusting activities from the total number of Local IDSs (LIDSs), *E*, as calculated in (1), each one installed on a node of the network. The set of the *m*_*i*_ LIDSs included in the *i*th cluster of the network is *L*_*i*_ = {*L*_*i*1_,*L*_*i*2_,…,*L*_*im*_}, sketched in Fig. 1.

(1) $$E = \sum\limits_{i = 1}^n \left( {{m_i}} \right)$$

The notation adopted in this paper is summarized in Table 2.

**Table 2:** Notation used in this paper.

Notations	
*C*	Set of *n* clusters
*C*_*i*_	The *i*_*th*_ cluster
*m*_*i*_	The number of nodes in the *i*_*th*_ cluster
*N*_*i*_	The set of nodes for the *i*_*th*_ cluster
*N*_*im*_	The *m*_*th*_ node of the *i*_*th*_ cluster
*E*	The total number of LIDSs in a WSN
*L*_*i*_	The total number of *m*_*i*_ LIDSs in the *i*_*th*_ cluster
*L*_*im*_	The *m*_*th*_ LIDS in the *i*_*th*_ cluster

A LIDS is a light version of a classical IDS adopted to run on a node of a sensor network. Its efficiency is absolutely affected and limited by the local memory, the battery power and all related characteristics inherent in a node of a sensor network. All these constraints prevent the direct employment of ordinary operating systems and applications on sensor nodes (Wittenburg & Schiller, 2006). Therefore, alternative OSs have been developed to support the special requirements of these devices, like the most common open source TinyOS (Hill et al., 2000), created at UC Berkeley. Likewise, special software design patterns have been introduced to be employed in the TinyOS (Gay, Levis & Culler, 2007). Moreover, specific applications implemented for WSNs use the C standard programming language. Such a decision has been implied as this is the actual language used for embedded systems that work under constraints in memory and power, and limited computational capabilities (Wittenburg & Schiller, 2006).

Figure 1 depicts a network of this architecture, with a sensor field of five clusters, and a ranging number of nodes included in each of them. Every node of a cluster communicates with all others in the same cluster and connects with the base station. The GIDS has been installed on the base station and twenty four LIDSs have been set up to work on the corresponding nodes.

## The game

Suppose a user is using the sensor network from a node of a cluster. He is a legitimate user and he has specific rights granted from the system, in accordance with its security policy. But the user is a potential attacker, who is acting normally and intrusively, depending on what goals he wants to achieve. When he is acting normally, the node looks as if it were a regular node of the network, misleading that it works properly.

Consider a case where this user requests resources from the network, breaching the security policy of the system. In other words, the user tries to get from the system resources but he has no right to do so. There are two possible reasons that this might happen; either the user has the right to get this resource but his request exceeds some specified limits, or the user tries to get resources but he is not authorized. In both situations, the user exploits the fact that he is a user of the system, and as so, he has some rights to use it. So, he is authorized for certain actions. Therefore, under these circumstances, it is a challenging task to draw a line that separates the user between normal and attacker.

A game with three players is constructed to model what described in Section The Architecture of the Model. The number of players has been decided and imposed by the architecture of the model (Fig. 1). In an extended version of the proposed game there might be four players or more that could trigger distributed attacks against a WSN. But in non-cooperative Game Theory, the more players play the game the more difficult it is to solve (Daskalakis, 2008; Daskalakis, Goldberg & Papadimitriou, 2009; Roughgarden, 2010). Daskalakis and Papadimitriou prove in Daskalakis & Papadimitriou (2005) that computing a Nash equilibrium in a three player game is a problem that belongs in the PPAD-complete class, a class defined to address the problem of finding a NE in polynomial time. Furthermore, they prove in Daskalakis, Goldberg & Papadimitriou (2005) that finding a Nash equilibrium in a 4-player game is also PPAD-complete.

In order to define the game, the following formal elements should be specified:

• **The list of *players*.** There are three players, the Potential Attacker (PA), the Local Intrusion Detection System (LIDS), and the Global Intrusion Detection System (GIDS).

• **Their possible *actions*.** PA’s strategy set includes two action types, the *normal action* type, and the *attacking action* type. LIDS’s actions are two, the *suggestion for suspicious* and the *suggestion for trusting*. The GIDS’s actions are also two, the *exclude* action and the *admit* action.

• **What the players know when they act.** When the game starts, no player has enough information, and thus they act under great uncertainty. PA might know that LIDSs and a GIDS monitor the WSN. The LIDSs and the GIDS keep history traces on how the game is being played, valuable information for next rounds.

• **The *outcomes* of the players’ actions.** The total number of the possible outcomes of the game is eight, which derives from all possible combinations between the three players’ actions (2 * 2 * 2).

• **The players *preferences* over these outcomes.** To quantify the outcomes of the game, first there is a need to specify preferences over outcomes. The von Neumann-Morgenstern utility function is used. A player prefers a strategy over another, because he gains more or he loses less. Following Binmore’s method (Binmore, 2007), numbers are assigned to reflect these preferences, and all players’ utility functions are constructed. Next, 0 is set to the least preferred strategy and 1 to the most preferred strategy. Using rational numbers, a value is assigned to every strategy, according to corresponding rankings. Then, the values free of fractions are obtained, after multiplying with their least common factor. The results are the payoffs of the game.

The first player is a user of the system who is a Potential Attacker (PA) under certain circumstances, namely a node of the sensor network. It is assumed that the PA is anyone excpet the system administrator, because in this situation, the user could be fully authorized and no distinction can be easily made between *normal action* and *attacking activity*. The second player is the Local IDS (LIDS), hosted by the node responsible for making suggestions for *suspicious* or *trusting* activities, triggered by the PA. Finally, the third player is the Global IDS (GIDS) that resides in the base station of the network, takes into account the LIDS suggestions, examines the history of the corresponding node’s activities, and decides whether to *exclude* or to *admit* what the user has requested.

Summarizing, player PA has the two strategies *normal action* and *attacking action*, player LIDS has the two strategies *suggestion for trusting* and *suggestion for suspicious*, and player GIDS has the two strategies *admit* and *exclude* a user’s action as normal or intrusive respectively.

There are two main forms for the construction of a game that represents a problem; the normal and the extensive forms. In the normal form games players act simultaneously, whereas in extensive form games players act sequentially, the one after the other. We have chosen the extensive form for the construction of the proposed game model to represent appropriately the interactions that take place between an internal attacker, a LIDS and the GIDS (Osborne, 2004).

**Figure 2:**
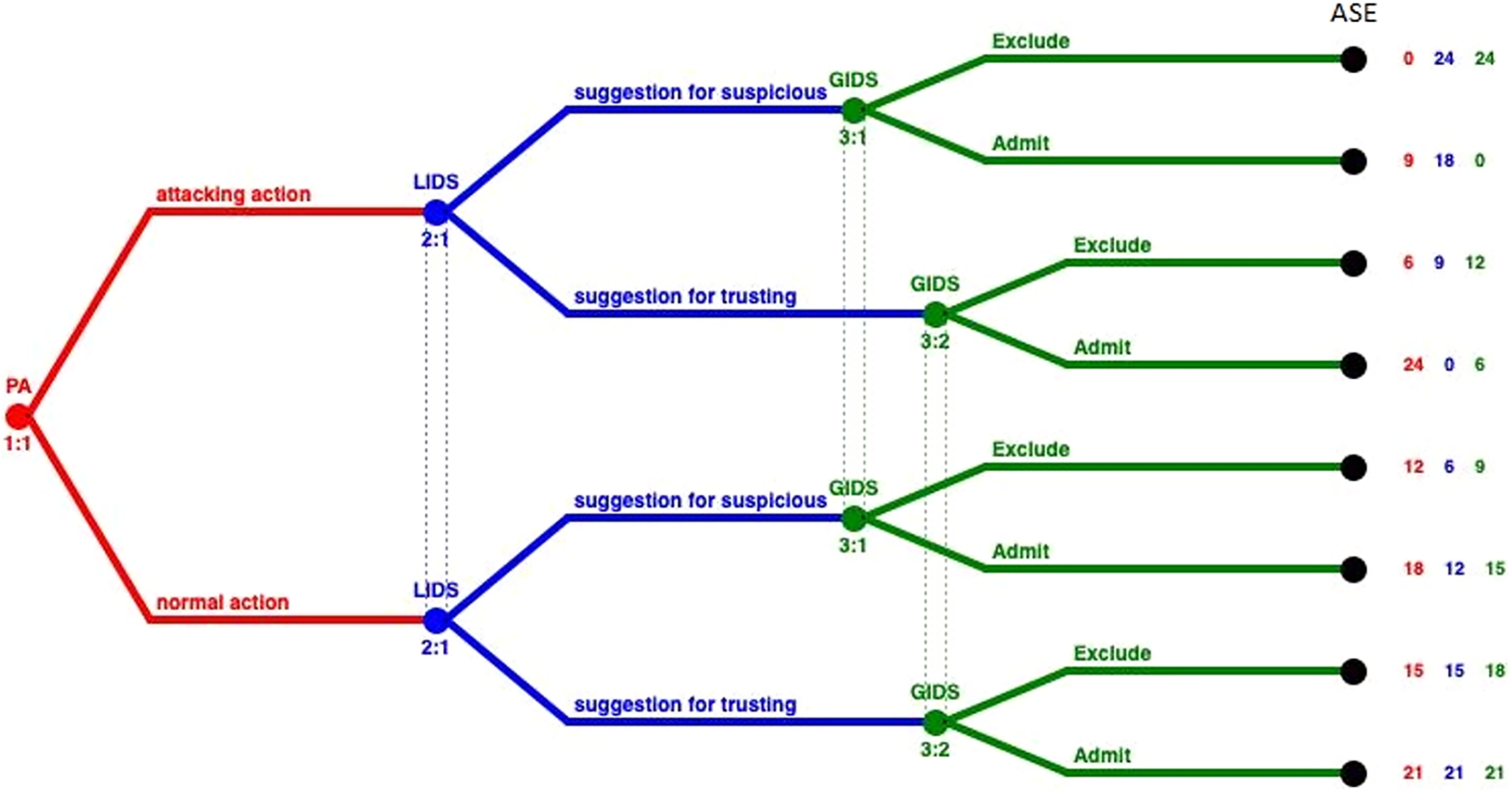
GoWiSeN in an extensive form game.

Figure 2 represents the extensive form of the GoWiSeN. Extensive form games are portrayed by trees. Player *PA* moves first at the initial node (the root) of the game, denoted by a red circle. The player’s name is displayed above the node. Below the node, the default labeling is the information set’s number. It is a unique identifier of the information set, in the form *player number*:*information set number* (*e.g*. 1:1 means the first move of the first player, *i.e*. the first move of player *PA*). Von Neumann defined information sets to model the progressive learning of which decisions will actually be made (Binmore, 2007).

Similarly, player *LIDS*’s moves start at blue circles, above which *LIDS* is written, and below, a corresponding pair, labeling its information set’s number (2:1 means that the second player, player *LIDS*, moves for the first time).

It is assumed that players *LIDS* and *GIDS* are not totally certain that player *PA* has chosen one of the actions included in his action set. This is consistent with the hypothesis that there is no detection engine with 100% detection rate. Thus, player *LIDS*’s (or *GIDS*’s) sub-trees belong to the same information set, connected with a dotted line to indicate this. In short, the dotted line connects player *LIDS*’s (or *GIDS’s*) nodes to indicate the Local or the Global IDS accuracy respectively, and thus, the degree of uncertainty whether player *LIDS* (or *GIDS*) has chosen a (*suggestion for suspicious*) or (*suggestion for trusting*) action (or an (*Exclude*) or (*Admit*) action).

Looking at the ends of the branches, 8 outcomes are identified. The number of outcomes derives from all the possible combinations between the PA’s actions and the Local and Global IDSs’ actions (2^3^). There are three capital letters at the end of the upper branch of the tree only and above the node as an example, that denotes player *PA*’s, player *LIDS*’s, and player *GIDS*’s choices, respectively. Specifically, (*ASE*) means that player *PA* has chosen an (*attacking*) action, player *LIDS* followed with a *suggestion for suspicious* action, and player *GIDS* chose afterwards to *Exclude PA*’s action.

Finally, the tuple of three numbers next to each end node is the players’ payoffs, that is to say, the outcome a player receives when a certain action has been chosen, represented as a number. The outcomes of the game are quantified, by first specifying preferences over outcomes, and then by using the von Neumann-Morgenstern utility function (Binmore, 2007). The red number belongs to player *PA*, the blue number belongs to player *LIDS*, and the green number belongs to player *GIDS*.

The players play the game repeatedly an infinite number of times. The reason is that, the user is not a random attacker, but an internal user of the system, who spends a long time every day in front of it. We assume him as a traitor rather than a masquerader. In repeated games, the actions are called strategies to distinguish them from the actions in the stage game.

## Solution of the game

In noncooperative game theory, the NE is the most commonly used solution concept. A Nash equilibrium of a game is a set of players decisions that results in an outcome, such that, no player has any reason to deviate from his choices, given that all the players do the same. John Nash proved that every noncooperative game has at least one Nash equilibrium (NE) (Nash, 1950; Holt & Roth, 2004). When no NE exists in pure strategies, then there is at least one in mixed strategies. In games with more than one NE, the problem of multiple NE and which one to choose appears (Osborne, 2004).

We proceed to solve the game as a three player game. A three player game obviously is more complicated than the two player games with a more complex tree. For each round of the game, the tree has three different levels. This increases the size of the tree, which makes the solution of the game difficult to be located.

In a three player game, where each player has a limited number of strategies, a matrix can be used. The matrix should have three dimensions with the third dimension devoted to the third player strategies. In practice, this is easily accomplished with an ’add pages’ technique (Dixit & Skeath, 1999). The first page of the matrix depicts the payoffs of the first strategy of the third player. The second page of the matrix depicts the payoffs of the second strategy of the third player, etc. Tables 3 and 4 depict the two pages of the matrix that describes the GoWiSeN game, which correspond to the two strategies PA might choose, the *attacking action* or the *normal action* respectively. Each page has two rows for player LIDS’ strategies (*suggestion for suspicious* and *suggestion for trusting*) and two columns for player GIDS’ strategies (*Exclude* and *Admit*). The two pages follow one another to represent the same instance. There is a pair of three numbers in each cell, which correspond to the payoffs of the PA, the LIDS, and the GIDS respectively.

**Table 3:** 1st page of the matrix-Attacking action strategy of PA.

PA chooses an attacking action			
		Global IDS	
		Exclude	Admit
Local IDS	Suggestion for suspicious	0, 24, 24	9, 18, 0
	Suggestion for trusting	6, 9, 12	24, 0, 6

**Table 4:** The 2nd page of the matrix-normal action strategy of PA.

PA chooses a *normal action*			
		Global IDS	
		Exclude	Admit
Local IDS	Suggestion for suspicious	12, 6, 9	18, 12, 15
	Suggestion for trusting	15, 15, 18	21, 21, 21

### Removing dominated strategies

We first solve the GoWiSeN game by applying the domination criterion, which says that a rational player should not use a dominated strategy. Binmore (2007) expresses the domination criterion by assuming two strategies *s*_1_ and *s*_2_ of a player *I* and three strategies *t*_1_, *t*_2_, and *t*_3_ of a player *II*. Then we decide that for player *I*, strategy *s*_2_ strongly dominates strategy *s*_1_ when

(2) $${\pi _1}({s_2},t) > {\pi _1}({s_1},t)$$

for all three values of player *II*’s strategy *t*. Moreover, if the relation between two strategies is ≥, then the one strategy weakly dominates the other.

In our game we express in algebraic terms the above criterion to check if it holds. First, we consider that player *PA* chooses an *attacking action* (Table 3). In this case, given that player *GIDS* chooses *Exclude*, player *LIDS* will choose strategy *suggestion for suspicious*, which dominates *suggestion for trust*, because 24 > 9. Similarly, given that player *GIDS* chooses *Admit*, player *LIDS* will choose again strategy *suggestion for suspicious*, which dominates *suggestion for trust* too, because 18 > 0.

Using this domination argument, we remove strategy *suggestion for trust* from the payoff matrix of Table 3 and the matrix changes to the following (Table 5):

**Table 5:** The 1st page of the matrix-attacking action strategy of PA, altered by removing strategy *suggestion for trust*.

PA chooses an attacking action			
		Global IDS	
		Exclude	Admit
Local IDS	Suggestion for suspicious	0, 24, 24	9, 18, 0

Reversing the above reasoning, we consider that player *LIDS* chooses *suggestion for suspicious*. Then, player *GIDS* will choose strategy *Exclude*, which dominates *Admit*, because 24 > 0. Similarly, given that player *LIDS* chooses *suggestion for trusting*, player *GIDS* will choose again strategy *Exclude*, which dominates *Admit* too, because 12 > 6.

Thus how we reduce the payoff matrix again by removing strategy *Admit* which is dominated by strategy *Exclude*. The payoff matrix now has the following form (Table 6):

**Table 6:** The 1st page of the matrix-attacking action strategy of PA, altered by removing strategy *Admit*.

PA chooses an attacking action		
		Global IDS
		Exclude
Local IDS	Suggestion for suspicious	0, 24, 24

Consequently, the above deletions lead to the conclusion that player *GIDS* will prefer to choose strategy *Exclude* regardless what player *LIDS* chooses. Another conclusion is that both players, the *LIDS* and the *GIDS*, have dominant strategies, which is indispensable precondition for three player games to have equilibrium. Therefore, in this subgame there is a unique equilibrium, the (*attacking action*, *suggestion for suspicious*, *Exclude*) = (0, 24, 24).

Finally, we examine the above equilibrium, if it is a Nash equilibrium or not. A Nash equilibrium must hold that no players have interest to leave the equilibrium and select another strategy. In this three player game, we check whether players *LIDS* and *GIDS* would choose other strategies than those located at the equilibrium. If player *LIDS* chooses strategy *suggestion for suspicious*, then player *GIDS* will choose strategy *Exclude* as the most beneficial (24 > 0). Conversely, if player *GIDS* chooses strategy *Exclude*, then player *LIDS* will choose strategy *suggestion for suspicious* as the most beneficial (24 > 9). Obviously, no one between players *LIDS* and *GIDS* has any interest to leave the equilibrium (*attacking action*, *suggestion for suspicious*, *Exclude*). Therefore, this equilibrium is a Nash equilibrium.

Then, we check if the domination criterion expressed in Eq. (2) holds, when considering that player *PA* chooses a *normal action* (Table 4). Following the same reasoning, we conclude that player *GIDS* will prefer to choose strategy *Admit* regardless what player *LIDS* chooses. Another conclusion is that both players again, the *LIDS* and the *GIDS*, have dominant strategies, which is indispensable precondition for three player games to have equilibrium. As a result, in this subgame there is a unique equilibrium where all players receive 21 each. This equilibrium is very beneficial for player *PA*, because he gets his highest payoff of the game (payoff 21). The equilibrium is the (*normal action*, *suggestion for trusting*, *Admit*) = (21, 21, 21).

We now examine the last equilibrium, if it is a Nash equilibrium or not, in a similar way as we did for the first page of the matrix of the game. We check whether players *LIDS* and *GIDS* would choose other strategies than those located at the equilibrium. If player *LIDS* chooses strategy *suggestion for trusting*, then player *GIDS* will choose strategy *Admit* as the most beneficial (21 > 18). Conversely, if player *GIDS* chooses strategy *Admit*, then player *LIDS* will choose strategy *suggestion for trusting* as the most beneficial (21 > 12). Obviously, no one between players *LIDS* and *GIDS* has any interest to leave the equilibrium (*normal action*, *suggestion for trusting*, *Admit*). Therefore, this equilibrium is also a Nash equilibrium.

Summarizing, the subgames equilibria located in the GoWiSeN game are two Nash equilibria; the (*attacking action*, *suggestion for suspicious*, *Exclude*) = (0, 24, 24) and the (*normal action*, *suggestion for trusting*, *Admit*) = (21, 21, 21). Decoding these findings we conclude that both equilibria are absolutely desirable for our model architecture described in Section The Architecture of the Model. In a case of the first equilibrium, although player *PA* attacks the network, both IDSs, the Local and the Global detect the attack and react properly. In a case of the second equilibrium, player *PA* behaves as a normal node of the network, the LIDS trusts its activity, and the GIDS permits this normal activity to be continued.

### Solving with gambit

Computing a Nash equilibrium is a fundamental problem in Algorithmic Game Theory (Nisan et al., 2007). Following Daskalakis and Papadimitriou proofs in Daskalakis & Papadimitriou (2005), computing Nash equilibria in a three player game is a problem that belongs in the PPAD-complete class, a class defined to address the problem of finding a NE in polynomial time. The complexity of computing a NE was consequently addressed in Daskalakis, Goldberg & Papadimitriou (2009) and Roughgarden (2010). The Gambit tool provides algorithms to compute NE in non-cooperative finite games (The Gambit Project, 2019).

In Removing Dominated Strategies, we located two Nash equilibria in the pure strategies of the GoWiSeN game. In order to locate also equilibria in mixed strategies, we use the Gambit tool (McKelvey, McLennan & Turocy, 2007). The Gambit tool runs different algorithms. The GoWiSeN is a three player extensive form game. The ‘Compute equilibria of a game using polynomial systems of equations’ algorithm was selected as the most suitable, following Gambit’ s documentation (The Gambit Project, 2019). The computational complexity of the PPAD class is thoroughly discussed in Nisan et al. (2007).

**Figure 3:**
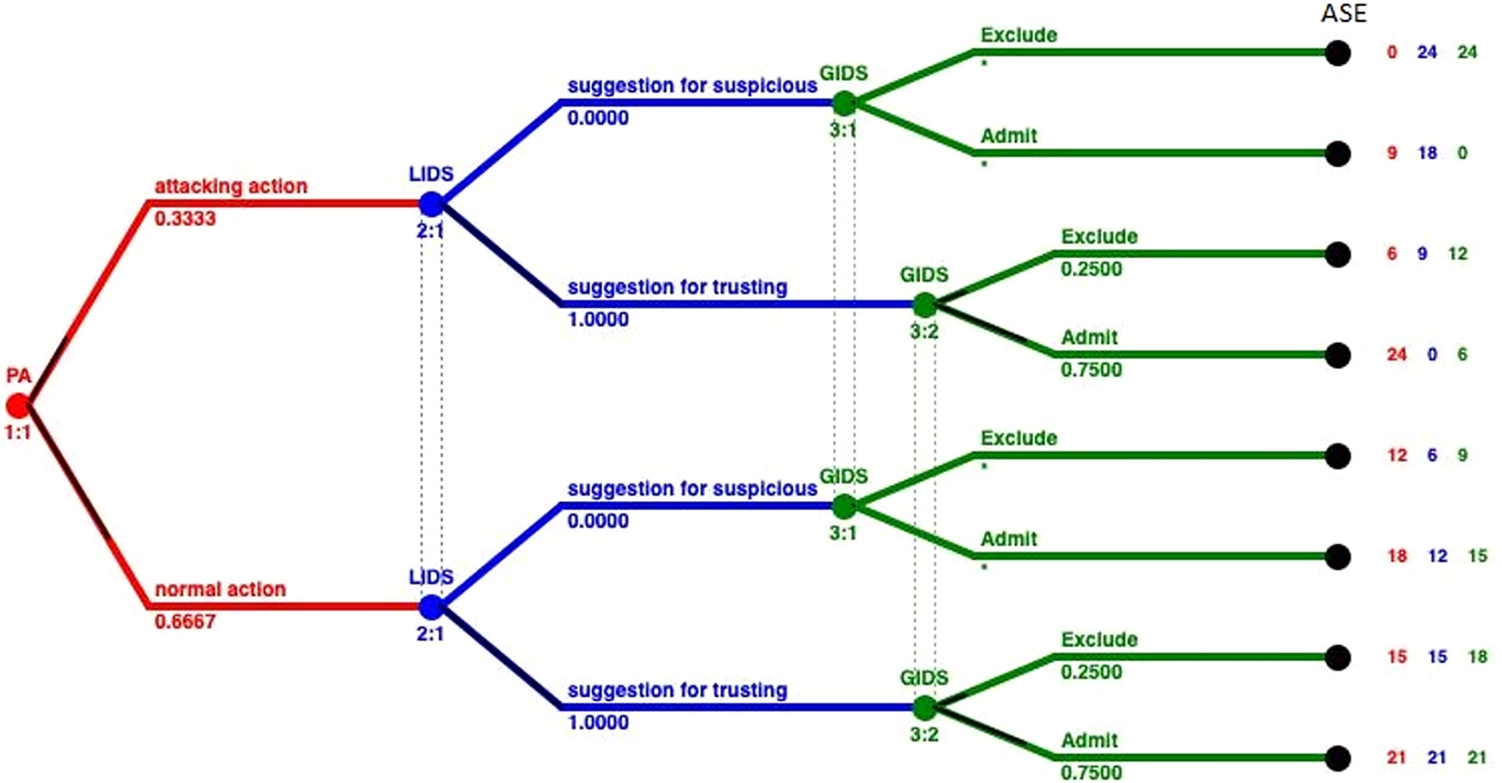
Mixed strategies of the GoWiSeN game located by the Gambit tool.

Solving the GoWiSeN game in extensive form with the Gambit tool we get one Nash equilibrium. This unique Nash equilibrium in mixed strategies indicates the existence also of a unique equilibrium in behavioral strategies. In Fig. 3 the solution of the GoWiSeN game reveals the mixed strategies.

Numbers below each branch (zeroes, ones, or others) indicate the solution of the game. Zero means that an action will not be chosen (probability 0), whereas one means that an action will be selected with certainty (probability 1). Likewise, 0.25 for instance is the probability the related action to be chosen. Assuming that the GoWiSeN game starts with player *PA* to have 1/3 probability to be an attacker and 2/3 probability to be a normal user, the Gambit tool shows that regardless an *attacking action* or a *normal action* has been chosen, player *LIDS* will definitely choose *suggestion for trusting* with probability 1. This is because the probability player *PA* to be a normal user is much higher than to be an attacker.

As for player *GIDS*, it excludes with probability 1 an *attacking action*, for which player *LIDS* gives with zero probability a *suggestion for suspicious*. This happens because payoffs have been calculated upon players’ preferences and thus they reflect their tendencies and first choices, which might totally conflict other players’ beliefs. In the same information set that matches the case of a normal user (3:1), player *GIDS* chooses *Exclude* with probability 1 when player *LIDS* has suggested suspicious activity. Next, in the second information set that matches the case of an attacker (3:2), player *GIDS* chooses *Exclude* with probability 0.25 or *Admit* with probability 0.75 regardless whether player *LIDS* has chosen *suggestion for suspicious* or *suggestion for trusting*.

## A case study

We have chosen a case study to confirm the functionality of the proposed game applied in fire fighting. WSNs can significantly assist the work of fire extinguishing, when they are working securely. Fire fighting is one of the most dangerous jobs, often with human victims. The risks associated with it are connected with several factors, as for example, the incomplete information about the exact location and the extent of a fire. The use of WSNs might reduce the number of risks associated with the firemen, and assist the quick and effective fire extinguishing. Finally, they might give additional information to the experts who investigate the cause of a fire, especially in cases where the fire has been caused intentionally.

### Wireless sensor networks for fire fighting

WSNs can be distinguished between data collection and event detection networks (Dutta, 2004). In those applications where the aim is the data collection, the sensors might be necessary to collect information every short periods at predefined time intervals of the day. As a consequence, for the rest of the time, the sensor node remains idle, so it saves power. However, in those cases where a WSN would be used for event detection, as it is the fire detection case, the sensor nodes should be alert, consuming continuously their power.

WSNs applied in fire fighting have special requirements that can be summarized as in the following (Sha, Shi & Watkins, 2006):

- False alarms must be kept to a minimum, because they consume time and resources of the fire brigade and thus might lead to unavailability of services in real instances.
- The WSN should be secure so that malicious activities must be deterred, because they might cause false alarms and send false information.
- As a fire might spread out quickly, the initial node that detects the event must send the data as soon as possible. If it fails to do so, then some alerts might not be set and valuable information might be lost. Moreover, this initial node must awake other adjacent nodes before destruction.
- The fire brigade must be connected with the WSN in order to exchange information.
- The network must be able to reroute its packages in cases of partial destruction, *i.e.*, when specific nodes are destroyed, to ensure the uninterruptible operation of the network, Therefore, a feature that allows the automatic adjustment of the routing table is required.
- The data transfer rate should be significantly high in order to keep information valuable and accurate.
- The fire brigade should know the exact positions of the sensor nodes.
- There must be a visualized demonstration of the location and spread of the fire as well as of the temperatures inside the building.
- The sensor nodes must be properly protected against high temperatures, to ensure their functionality and their ability to work accurately.

Among the described requirements, the proposed game mainly aims at fulfilling the first and the second one, by detecting intrusive activities and so reducing the number of false alarms raising by malicious nodes.

### The installation of a wireless sensor network in a building

**Figure 4:**
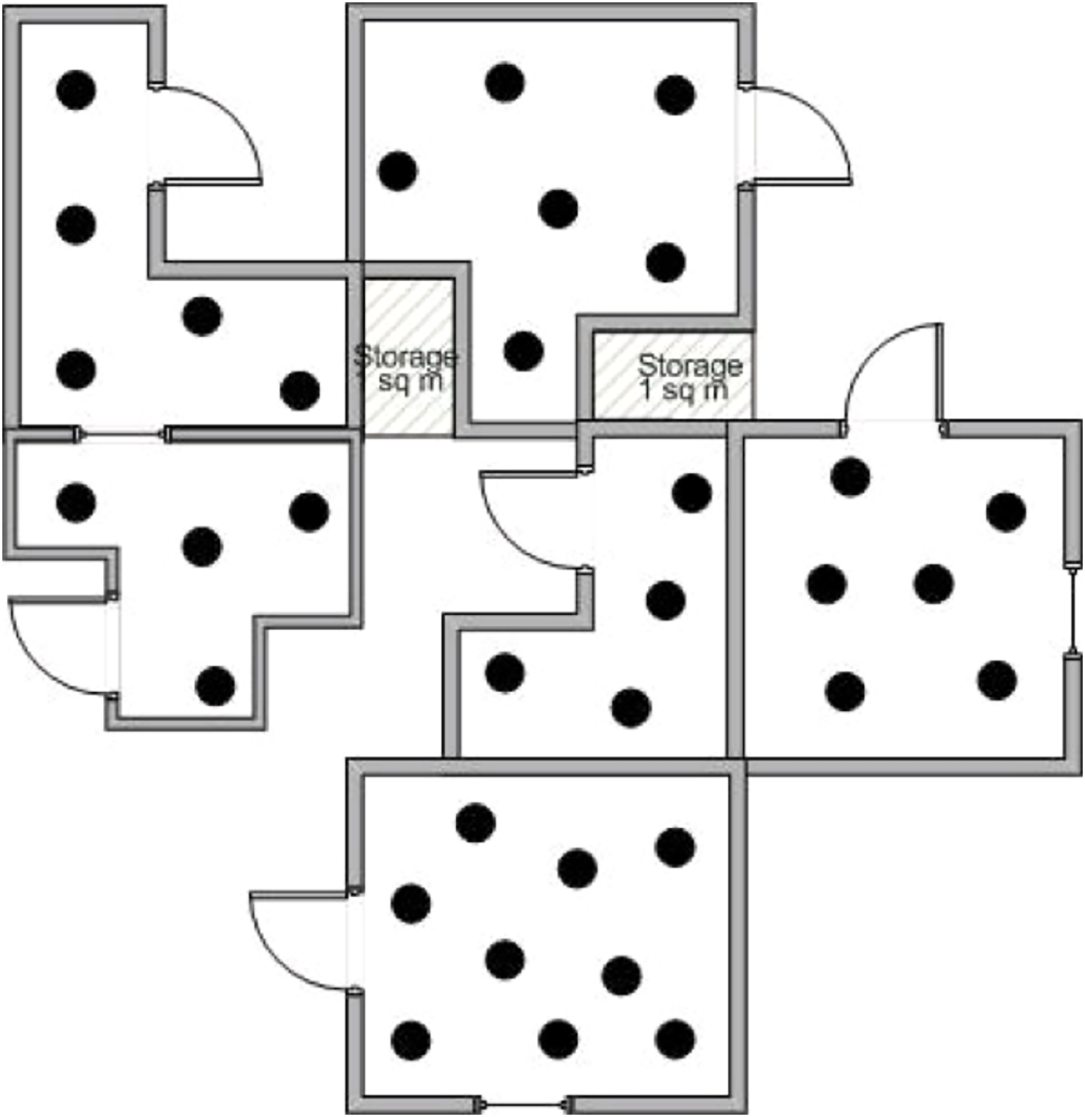
A division of the area covered by the WSN.

Consider a WSN installed throughout the rooms of a company’s headquarters in a flat of a skyscraper in NY. The whole area extents to 2,500 square meters, and numerous small rooms are shaped as offices, using special light separators. Figure 4 presents only a division of the area occupied by the company. There are small rooms, nodes are scattered densely in each room, and a base station is cited somewhere in a safe place of the network’s deployment region. Different types of sensor nodes have been used: temperature sensors for detection and tracking, smoke detectors for detection, infrared detectors for tracking and smoke and movement detectors too.

The WSN is always connected with the fire brigade *via* the internet, so that, it could send an emergency signal in case of fire detection. As a result, when fire is detected, the director of the fire brigade will be able, *via* this connection, to send to firemen information related to the initial fire location and to the progress and direction of the fire. Moreover, when the firemen arrive at the fire location, they could connect to the WSN to receive the latest information about the fire spread. Detailed sketches that depict the building infrastructure and the node locations of the WSN will be helpful to the fire brigade.

Upon arrival, the firemen could use a portable computer in the place of the base station computer. Thus, as long as the nodes of the network are properly working, even if the base station has been destructed, the WSN will continue its functioning without problems.

### Implementing the GoWiSeN model

The safety of a WSN applied for fire fighting might be threatened by malicious unauthorized persons who try to gain access to the network. Next, such an intruder might intentionally cause a false alarm, or even alter information collected from the sensors of the network, affecting the validity and integrity of the information being transmitted to the base station.

The GoWiSeN model has been designed to protect a WSN of similar types of attacks. For this purpose, a Local Intrusion Detection System will be installed over each sensor node, named Local IDS. This LIDS is a light version of an IDS, with minimum power requirements. Furthermore, the base station will host a complete IDS package, named Global IDS (GIDS).

The GIDS keeps history records for all the events that have been identified as illegal activities of the network nodes. Consequently, a list is maintained for each node, rating from 0 to 5 the number of times the corresponding node has acted illegally. In particular, the value of 0 indicates a node that operates normally and has never been detected for malicious activities, whereas, the value of five denotes that the node has already been detected 5 times for attacking actions.

In addition, when a node communicates with other adjacent nodes, because it has a LIDS installed, it could examine their activities and preliminary conclude whether a node acts legitimately or not. Subsequently, it would send a signal of *suggestion for suspicious* or a signal of *suggestion for trusting* to the GIDS, depending on its conclusion. Finally, the GIDS on its turn, will decide whether it should *admit* or *exclude* the node’s activity.

A fire event is detected when some sensor nodes of the network detect temperatures higher than 40 °C for a longer period than 10 s. In case of fire, the LIDS will send a signal of *suggestion for trusting* to the GIDS, if the adjacent nodes report the same information. Under certain circumstances with high temperatures due to physical reasons, such as the phenomenon of heatwave during summer, the network manager should take care of the limits adjustment.

The GIDS should then examine three different parameters, in order to decide whether to admit or to exclude an activity suggested by a node. First, it should consider the LIDS’s suggestion either for trusting or for suspicious. Next, it should take into account the list that corresponds to the node reported the problem. As a final point, it might use the detection engine integrated in it, which uses classical detection techniques, to verify the initial findings and deduce a definite conclusion.

Two different scenarios demonstrate the network operation and functioning in the following subsections. Scenario A shows in detail the steps for fighting a real fire event, while scenario B presents the proposed model functioning, when an attacking activity, originated by a compromised node, takes place.

#### Scenario A

1. Suppose node *N*_21_ is a temperature detection sensor, belongs to cluster *C*_2_ and operates normally.
2. A fire event takes place somewhere inside the area occupied by the company’s headquarters. The fire point is next to node *N*_21_.
3. Node *N*_21_ detects temperature higher than 40 °C for a longer period than 10 s, and therefore, an alarm is automatically triggered.
4. The fire event detection is reported to the adjacent nodes.
5. The adjacent nodes also detect high temperatures according to the predefined thresholds, evaluate the received information, and they trigger alarms as well.
6. Node *N*_21_ sends a signal of fire event detection to the base station.
7. The adjacent nodes’ LIDSs send signals of *suggestion for trusting* to the base station.
8. The GIDS puts under consideration all the received information, examines the corresponding list with the node ranking, takes into account the adjacent nodes suggestions, and concludes to *admit* the reported event as real.
9. The base station sends all the collected data to the center of the fire brigade, the fire brigade estimates the current situation and counteracts against the fire.
10. When the operation has been completed, the fire brigade sends information to the GIDS regarding the accuracy of the initial information reported the problem.

#### Scenario B

1. Suppose node *N*_21_ is a temperature detection sensor, belongs to cluster *C*_2_ and operates normally.
2. A skilled intruder gains access to the WSN, by compromising node *N*_21_. His intentions include continuous attacks every short time periods. His aim is to set out of order a considerable part of the network, each time he completes an attack.
3. The compromised node *N*_21_ initiates an attacking activity. The attack is based on the flooding attack technique and will be carried out at the transportation layer of the network. The aim of the flooding attack is to exhaust the resources of the adjacent nodes, so that, a part of the network will be set out of use.
4. Each LIDS, that resides on an adjacent node, examines the data captured from this activity. Based on this data, the LIDS detects illegal activity, because the number of connection requests, from the compromised node *N*_21_, is unusually great. Therefore, all the LIDSs of the adjacent nodes will send signals of *suggestion for suspicious* to the base station, considering this activity as an attacking activity.
5. The GIDS puts under consideration all the received information, examines the corresponding list with the node ranking, takes into account the adjacent nodes suggestions, and activates the anomaly detection technique module to handle the unexpected operation. The module detects that the number of requests for connections is over the upper threshold, and the GIDS concludes to *exclude* the requested connections as malicious activities, preventing the network flooding.
6. The GIDS updates the list assigned to the compromised node, by increasing its ranking by 1. Then it checks if the number of times the node has been acted illegally is already 5, and if this is true, the GIDS adds the compromised node *N*_21_ into a blacklist, for all the routing tables of the network.

#### Applying the GoWiSeN model

Applying the GoWiSeN model should reveal the way the game described and solved in sections The Game and Solution of the Game would be played, in order to protect the WSN established for fire fighting.

In Scenario A, player *PA* is a normal user of the system, the user of node *N*_21_, which works normally. A fire event that takes place very close to this node, triggers an alarm by this node, which also sends information to the adjacent nodes. In GoWiSeN, player *PA* has chosen *normal action*. Afterwards, the adjacent nodes detect the event and evaluate the information received from node *N*_21_. Since they conclude that the information is valid, they recommend suggestion for trusting to the base station. This means that the main LIDS as well the adjacent LIDS will play *suggestion for trusting*, also because this strategy has the highest payoff regardless what the GIDS will choose.

The base station evaluates all the information collected by the related LIDS and estimates what the real situation is. Considering that the ranking of node *N*_21_ is low (its value is 0) and taking into account what the adjacent nodes recommend, player *GIDS* chooses *Admit*. This strategy also gives the best payoff to player *GIDS*, no matter what player *LIDS* plays. It is the Nash equilibrium located in Section Removing Dominated Strategies, (*normal action*, *suggestion for trusting*, *Admit*) = (21, 21, 21).

In Scenario B, player *PA* is an internal attacker of the system, the user of node *N*_21_, which is compromised. We assume that node *N*_21_ might work normally most of the times, but sometimes it is used by the insider to attack periodically the system. The insider aims at causing unavailability over a significant part of the network each time he accomplishes an attack. In such a case, the compromised node attacks the system. This means that player *PA* chooses *attacking action*. Any adjacent node and its *LIDS* that accepts the attack evaluates this move. The LIDS detects unusual behavior, because the compromised node suddenly starts sending a large number of connection requests. Player *LIDS* will choose *suggestion for suspicious* and will send the relevant information to the base station. Nevertheless, this strategy has the highest payoff.

In the base station, the GIDS examines the event and chooses *Exclude* preventing its completion. This decision is based also upon an anomaly detection technique which detects the unusual behavior of the aforementioned node, the history and the list with the rankings and updates the corresponding ranking for the compromised node *N*_21_ by adding 1. If the node ranking will be 5, then it becomes blacklisted for all the routing tables of the network. In this case, player *GIDS* will play *Exclude* regardless what player *LIDS* chooses. It is the Nash equilibrium located in Section Removing Dominated Strategies, (*attacking action*, *suggestion for suspicious*, *Exclude*) = (0, 24, 24).

## Experimental evaluations

After completing the theoretical analysis as presented in the previous sections, we decided to conduct simulations on realistic datasets using IDSs with different capabilities.

### Dataset

We use a recent dataset that was collected from a realistic network environment, namely the Bot-IoT dataset (Koroniotis & Moustafa, 2021) for the experiments. Table 7 summarizes the statistics of attacks in Training and Testing datasets. Both datasets satisfies the eleven indispensable characteristics of a valid IDS dataset, namely Anonymity, Attack Diversity, Complete Capture, Complete Interaction, Complete Network Configuration, Available Protocols, Complete Traffic, Feature Set, Metadata, Heterogeneity, and Labelling (Gharib et al., 2016). Table 8 gives a synopsis of the main simulation parameters that affect the experimental evaluations.

**Table 7:** Attack types in the Bot-IoT dataset.

Category	Attack type	Flow count	Training	Test
BENIGN	BENIGN	9,543	7,634	1,909
Information gathering	Service scanning	1,463,364	117,069	29,267
	OS Fingerprinting	358,275	28,662	7,166
DDoS attack	DDoS TCP	19,547,603	1,563,808	390,952
	DDoS UDP	18,965,106	1,517,208	379,302
	DDoS HTTP	19,771	1,582	395
DoS attack	DoS TCP	12,315,997	985,280	246,320
	DoS UDP	20,659,491	1,652,759	413,190
	DoS HTTP	29,706	2,376	594
Information theft	Keylogging	1,469	1,175	294
	Data theft	118	94	24
Total	/	73,370,443	5,877,647	1,469,413

**Table 8:** Simulation parameters.

Number of Players	3
Number of Nodes [Bot-IoT dataset]	8
Mean Simulation Time [Training & Testing]	1,220 [KMeans], 760 [Naive Bayes]
Actions	Alarm-Exclude\Admit

The BoT-IoT dataset contains more than 72,000,000 records devised on 74 files, each row having 46 features. We use the version proposed by Koroniotis et al. (2019), which is a version of training and testing with 5% of the entire dataset.

### Clustering and classification techniques

Assuming that the architecture described in Fig. 1 consists of IDSs of different capabilities due to power, storage and computational constraints, we decided to evaluate the performance of different IDSs on Bot-IoT dataset.

IDS performance is evaluated based on its capability of classifying network traffic into a correct type. Table 9, also known as confusion matrix, shows all the possible cases of classification.

**Table 9:** Confusion matrix.

		Predicted class	
		Negative class	Positive
Actual class	Negative Class	True negative (TN)	False positive (FP)
	Positive Class	False negative (FN)	True positive (TP)

We decided to use an IDS with poor performance to play the role of the LIDS and a classifier with very good performance as the GIDS. This is due to the fact that each simple node can play the role of LIDS and we cannot expect it to have high computation or energy capabilities and this we need to have a simple method running on it. On the same time GIDS is a specific node that can have additional capabilities. For LIDS we have used Kmeans clustering and for the GIDS the Naive Bayes method. The overall performance of both methods is presented in Table 10.

**Table 10:** Evaluation of different IDSs.

Method	True positive (TP)	False negative (FN)	True negative (TN)	False positive (FP)
Naive Bayes	0.999972	0.000028	0.975	0.025
Kmeans	0.55	0.45	0.916	0.084

**Figure 5:**
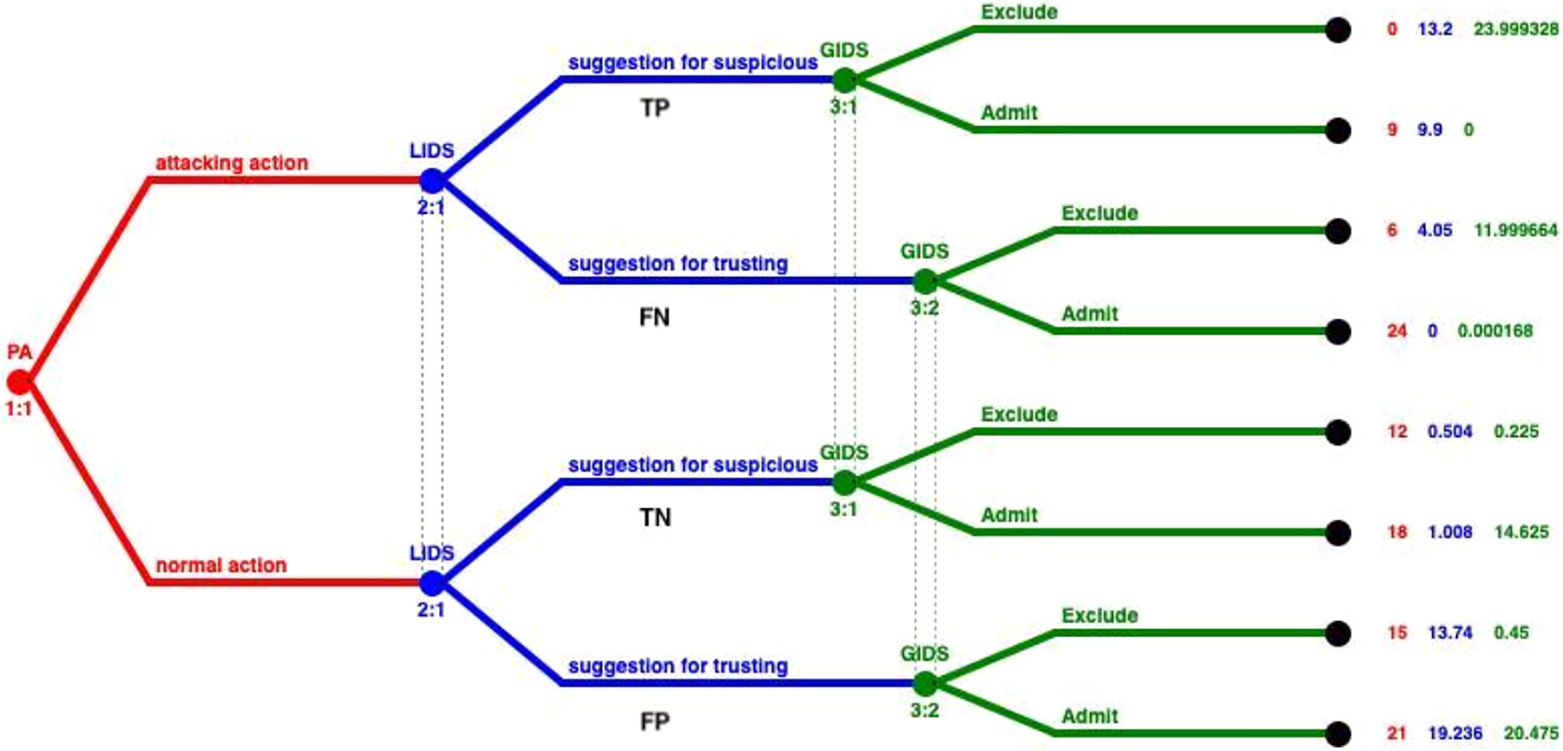
Integrating performance of IDSs in mixed strategies.

The aforementioned values correspond to the different arcs in the tree that is presented in Fig. 3. The pairing of each value of the IDS and the arcs on the tree is represented in Fig. 5. Based on this pairing, we changed the payoff of each player for every strategy, by assigning the payoff proportion, which corresponds to the probability a strategy profile being selected, as shown in Table 11. These probabilities derive from the detection rates provided by the dataset, and complies with the game theoretic solution approach followed to locate NE in mixed strategies (Bhuse, Gupta & Al-Fuqaha, 2007). Based on these calculations, the payoffs that incorporate the performance of the IDSs are inserted into the model, as shown in Fig. 5.

**Table 11:** Recalculated rewards for each player.

State	LIDS performance	GIDS performance	LIDS initial reward	GIDS initial reward	LIDS final reward	GIDS final reward
TP–TP	0.55	0.999972	24	24	13.2 5	23.999328
TP–FN	0.55	0.000028	18	0	9.9	0
FN–TP	0.45	0.999972	9	12	4.05	11.999664
FN–FN	0.45	0.000028	0	6	0	0.000168
FP–FP	0.084	0.025	6	9	0.504	0.225
FP–TN	0.084	0.975	12	15	1.008	14.625
TN–FP	0.916	0.025	15	18	13.74	0.45
TN–TN	0.916	0.975	21	21	19.236	20.475

**Figure 6:**
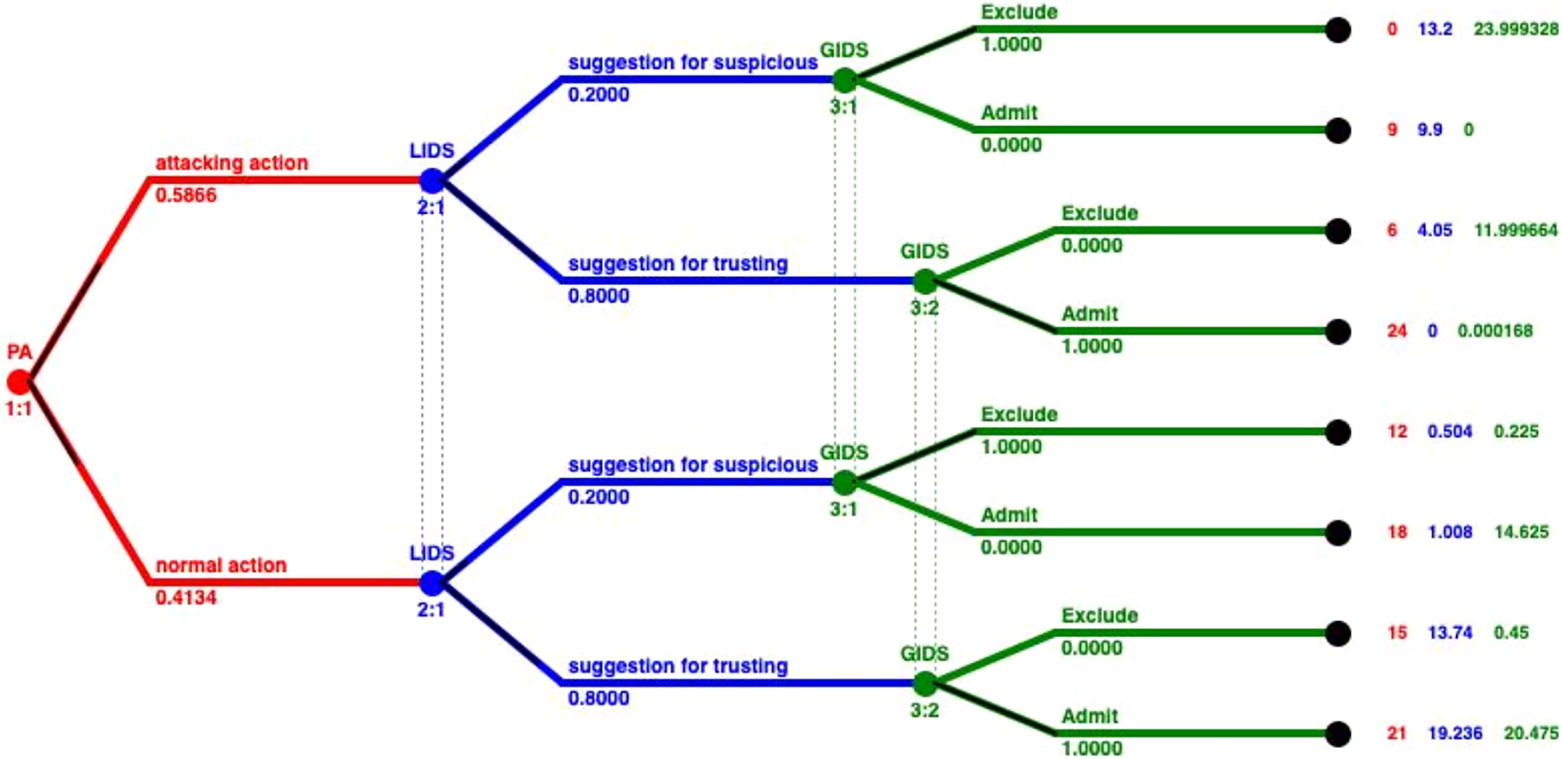
Mixed strategies with evaluation metrics of IDSs.

We solve again the model with the Gambit tool (McKelvey, McLennan & Turocy, 2007) and we get one Nash equilibrium. This unique Nash equilibrium in mixed strategies indicates the existence also of a unique equilibrium in behavioral strategies. In Fig. 6 the solution of the GoWiSeN game reveals the mixed strategies.

Numbers below each branch (Zeros, ones, or others) indicate the solution of the game. Zero means that an action will not be chosen (probability 0), whereas one means that an action will be selected with certainty (probability 1). Likewise, 0.20 for instance is the probability the related action to be chosen. By incorporating the evaluation metrics of the IDSs, we see that the probabilities in each branch has changed, representing a more realistic scenario, without deterministic decisions that dominated the previous model. We can observe that GIDS takes a on/off decision following the decision of the LIDS. LIDS on the other hand has probability 0.2 to fire an alarm and 0.8 to accept the bahavior of the node as normal, regardless of the action of the PA.

## Special cases

Except from the general case where one PA is performing in a malicious way and the LIDS is detecting this, there are some special cases that need to be modeled in a different way. These two special cases are presented in the following subsections.

### Overlapping clusters

In WSNs clustering is an efficient approach used to achieve optimal performance of the network. In traditional clustering methods disjoint clusters are created, using some specific criteria that include distance, energy, delay etc. On the other hand, many scholars have highlighted the significant advantages of creating overlapping clusters when applying intercluster routing, node localization, and time synchronization protocols (Youssef, Youssef & Younis, 2009; Maglaras & Katsaros, 2011; Abbasi & Younis, 2007).

**Figure 7:**
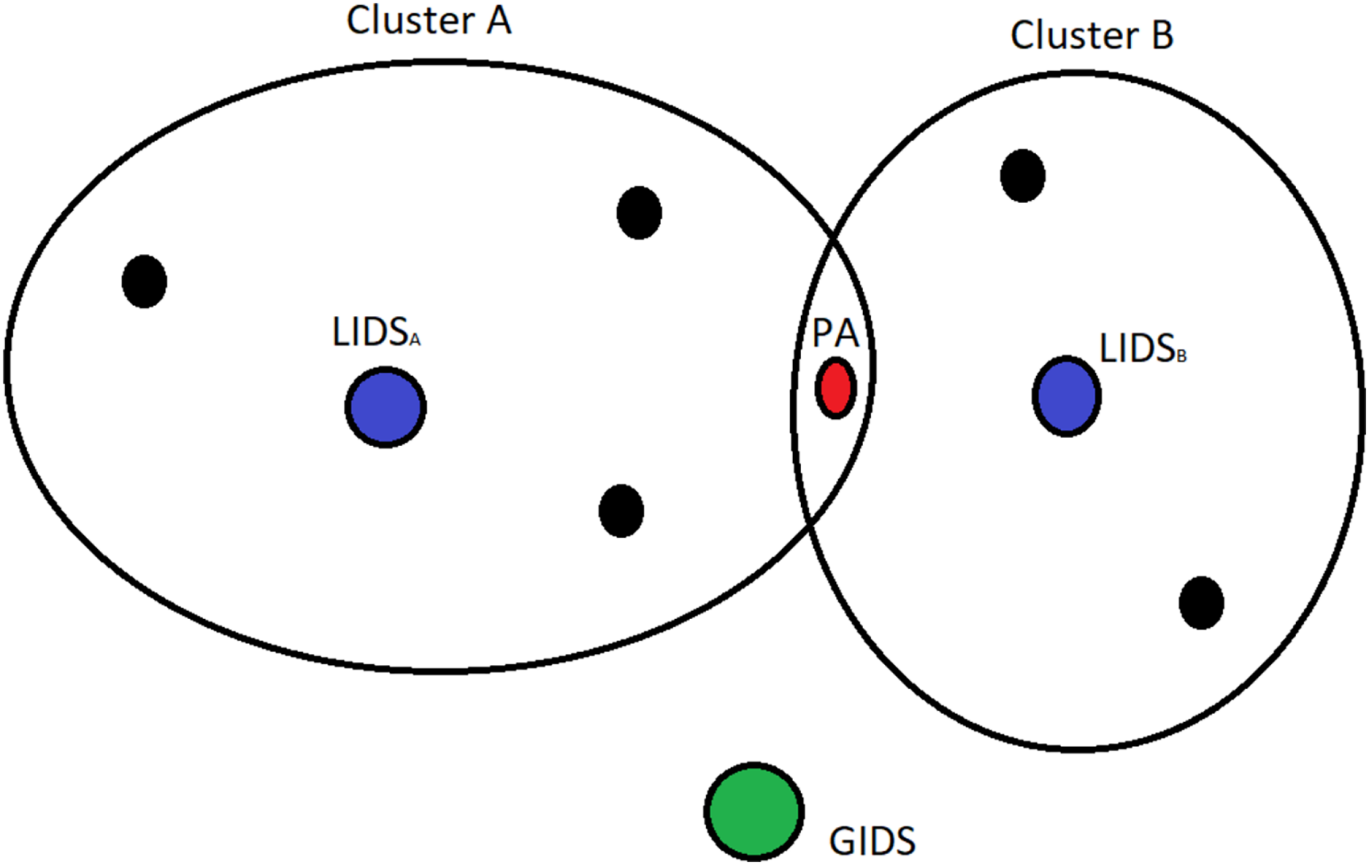
Special case of a PA that belongs to two clusters.

**Figure 8:**
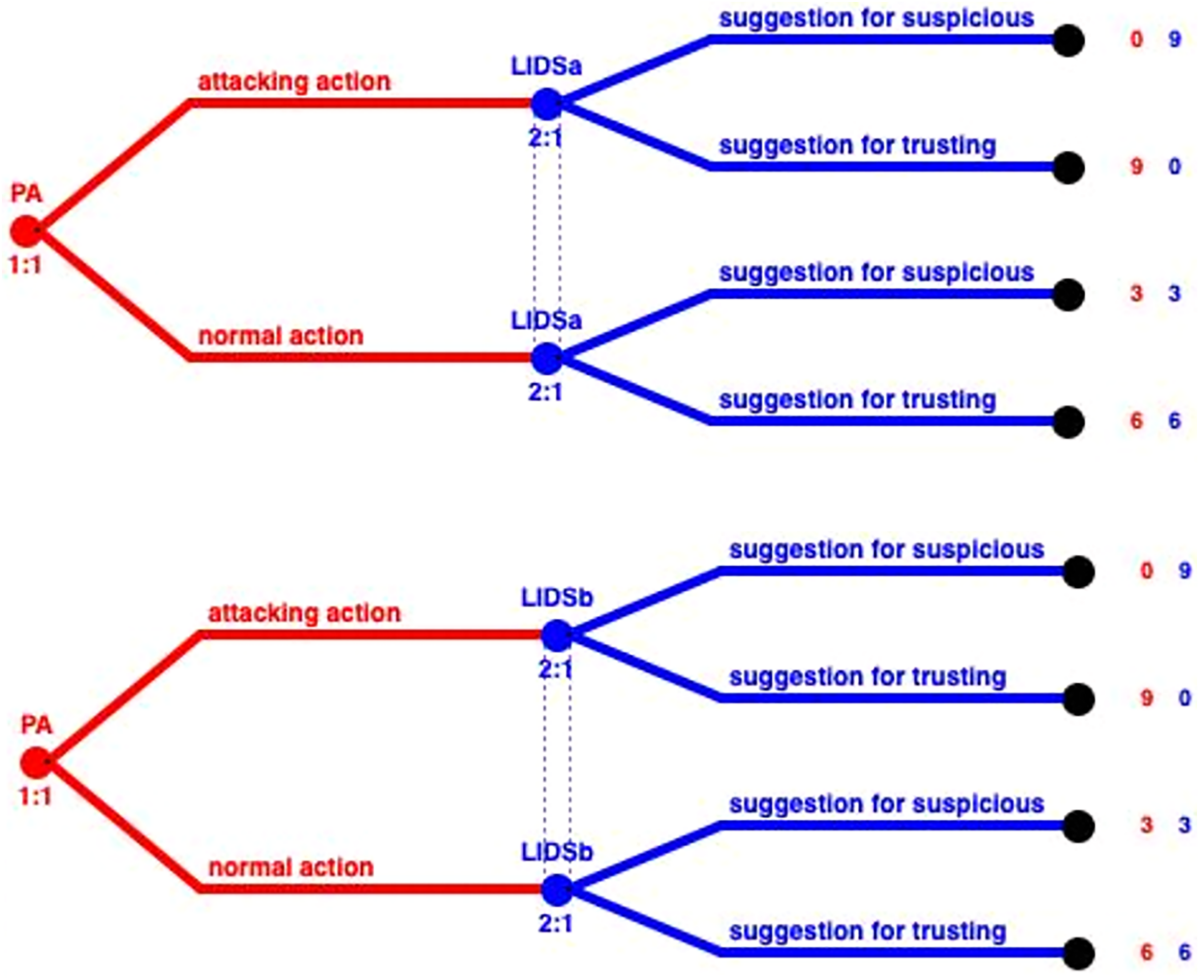
Subtrees of LIDSa and LIDSb.

In this case, several PAs belong to two clusters and thus are monitored by two LIDSs as represented in Fig. 7. In order to model this situation we need to construct different games. In Fig. 8, we can see the representation of a game that consists of the PA, that belongs to clusters A and B, and LIDSa of cluster A (left part of the figure). On the right part of the figure, in case these two IDSs perform the same, thus having the same TP, TN, FP, TN, these two short trees can be merged creating the same outcome as the normal case. In the occasion that these IDSs have different performance due to running different detection algorithms, or due to other parameters that affect the observation capabilities (distance, interference or even trust levels) these two trees cannot be merged, and the outcomes of the two LIDSs must be combined using smart techniques (Maglaras, Jiang & Cruz, 2016).

### LIDS under attack

During the analysis and evaluation of the system we have assumed that the LIDSs are immune to cyber attacks. This assumption is not very realistic since any node can play the role of a LIDS. In the specific case where a LIDS is infected, the GIDS can detect the attack and report back to the system, in order to ignore its alarms and also appoint another node as LIDS for the cluster. The reappointment of the LIDS can be done similar to a reclustering procedure, where nodes, through voting, elect the node that will play this role for their cluster (Behera et al., 2019).

**Figure 9:**
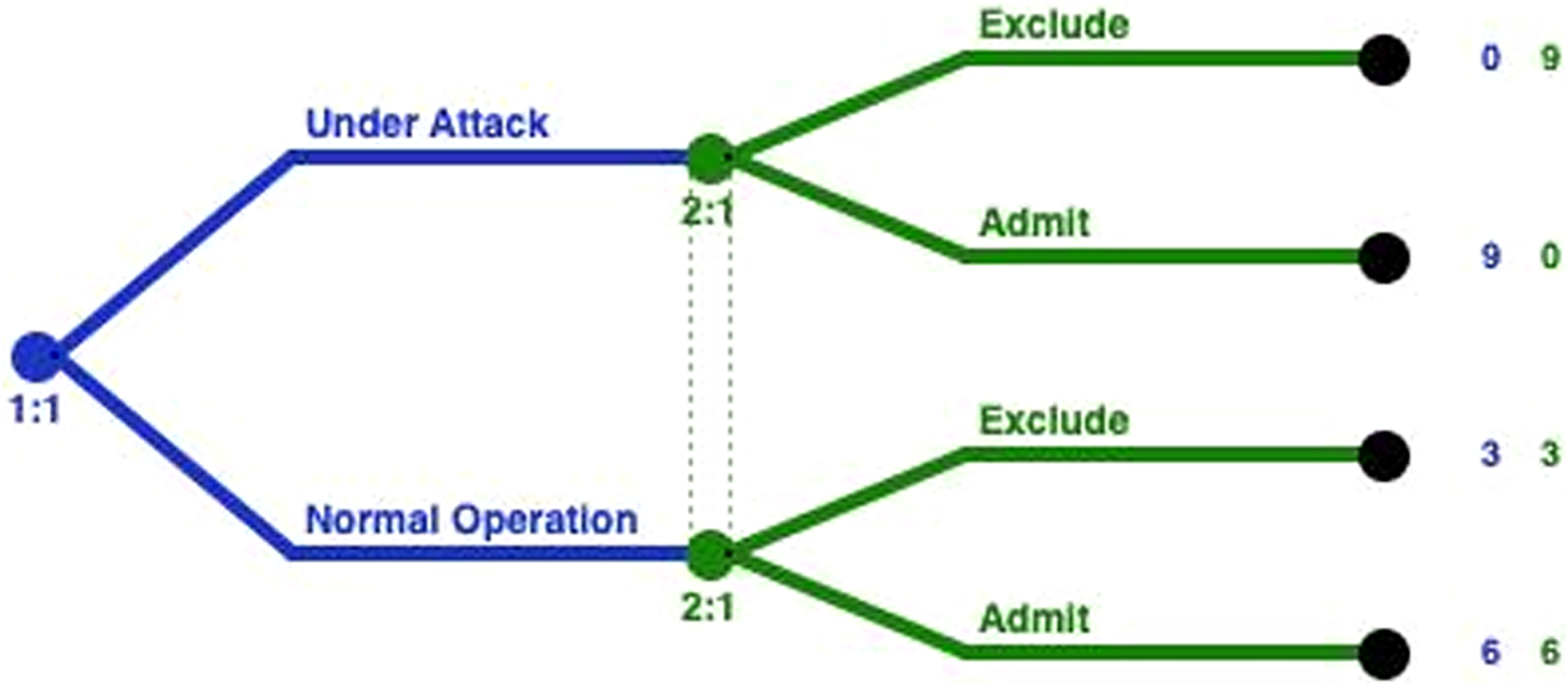
LIDS under attack model.

For this special case, the model that can be constructed consists of only two players, the LIDS (that plays the role of the PA) and the GIDS (that plays the role of the LIDS). Such a model is represented in Fig. 9 and can be solved in a similar way as the general case, but with fewer players and less states.

## Conclusion and future work

The incorporation of a game theoretic approach in the area of Intrusion Detection to confront internal attackers in WSNs was selected. To address the problem of detecting compromised nodes, a three player non-cooperative game was modeled, between Local IDSs located on different sensor nodes (LIDSs), a Global IDS (GIDS) and an insider. Possible interactions between an insider and Intrusion Detection Systems used in a WSN were examined, and possible suggesting strategies were studied through the solution of the game, by locating Nash Equilibria in mixed strategies. The evaluation of the model using a realistic dataset from a network environment was decided, to challenge its ability to discriminate between normal and malicious nodes. In special cases, nodes that belong to overlapping clusters were considered and the corresponding model was studied. Finally, the situation where a LIDS is compromised was examined and different solution approaches to this problem too were discussed. The results show how the game should be played, what the players choose to play and under which circumstances. An ultimate goal is the identification of a node as compromised and its exclusion of the network to prevent further damage.

The integration of the evaluation metrics of LIDSs and GIDS through the use of a realistic dataset further improves the efficiency of the proposed model. The use of different datasets, combination of more machine learning techniques and the use of smart ensemble methods can be considered as future research paths. Regarding a future extension of the theoretical game model, subgame perfect equilibria will be considered to improve collaboration between LIDSs and GIDS as well as different preference rankings that correspond to miscellaneous types of insiders.

## Acronyms

CPES: Cyber-Physical Embedded System
DDoS: Distributed Denial of Service
ESN: Embedded Sensor Network
FN: False Negative
FP: False Positive
GoWiSeN: Game of Wireless Sensor Networks
GIDS: Global Intrusion Detection System
IDS: Intrusion Detection System
IoT: Internet of Things
LIDS: Local Intrusion Detection System
NE: Nash Equilibrium
NY: New York
OS: Operating System
PA: Potential Attacker
TN: True Negative
TP: True Positive
VCG: Vickery-Clark-Grooves
WSN: Wireless Sensor Networks

## Additional Information and Declarations

### Data Availability

The following information was supplied regarding data availability: The data is available at figshare: Maglaras, Leandros (2021): Datasets. figshare. Dataset. https://doi.org/10.6084/m9.figshare.16627693.v1.
